# Effectiveness of advertising complaint mechanisms in South Africa: Public health implications and regulatory gaps

**DOI:** 10.1371/journal.pgph.0005223

**Published:** 2026-10-30

**Authors:** Sameera Mahomedy, Nosiphiwo Nzimande, Susan Goldstein

**Affiliations:** SAMRC/Wits Centre for Health Economics and Decision Science- PRICELESS SA (A Division of Wits Health Consortium), School of Public Health, Faculty of Health Sciences, University of Witwatersrand, Johannesburg, South Africa; Islamic Azad University South Tehran Branch, IRAN, ISLAMIC REPUBLIC OF

## Abstract

The prevalence of overweight and obesity among South African children is increasing. Unhealthy food and beverage marketing is a major contributor to this burden and has been associated with an increased risk of diet-related non-communicable diseases (DR-NCDs), unhealthy food preferences among children, and changes in household purchasing patterns. Despite these risks, South Africa primarily relies on industry self-regulation to govern advertising, raising questions about the effectiveness of existing complaint mechanisms. This study evaluated the procedural and substantive effectiveness of advertising complaint mechanisms for child-directed marketing across South African regulatory bodies. A ‘mystery shopper’ methodology was used to assess the complaint mechanisms under real-world conditions. Nine adverts were purposively selected from a broader observational sample, resulting in eleven complaints submitted to the relevant regulatory bodies. Complaints related to alcohol adverts, unhealthy food and beverage adverts, and promotional adverts across television, radio, YouTube and outdoor platforms. Complaint mechanisms were evaluated for timeliness, accessibility, jurisdictional clarity, and procedural and substantive adjudication. The current advertising framework is largely self-regulatory through the Advertising Regulatory Board (ARB), limiting the scope of enforceable oversight. Complaint procedures were frequently characterised by delays, incorrect referrals, inconsistent procedural requirements, and fragmented jurisdiction. Procedurally, nine of the eleven complaints were not satisfactorily resolved. Substantively, none were satisfactorily resolved. The ARB applied a narrow “specifically directed to children” test, despite broader evidence that children’s exposure to unhealthy product marketing can influence behaviour, particularly among adolescents. Comparison with relevant ARB decisions involving public health messaging further suggested asymmetries in evidentiary standards between commercial advertising and public health communications. These findings highlight important procedural and substantive limitations within South Africa’s current advertising complaints framework. They further support consideration of stronger statutory or co-regulatory approaches with independent oversight to better protect children from harmful commercial marketing.

## Introduction

South Africa has seen a rise in non-communicable diseases (NCDs) over the years, partly attributable to consumption of unhealthy food and beverages [1,2]. The burden of NCDs has more than doubled between 1990 and 2019, with morbidity and mortality shifting from communicable to non-communicable diseases, including obesity [3]. There has also been an increase of overweight and obesity amongst children, with obesity being classed as an NCD, as well as a risk factor for other NCDs [4]. In 2020, approximately 31% of children in South Africa had a high BMI, and with the second highest rate of increase globally this is predicted to rise to 71% in 2035 [5]. Obesity is a response to the modern food environment, where consumption of inexpensive, highly palatable, energy dense, nutrient poor food and beverages is highly prevalent [4]. These foods are aggressively marketed, including to children. Food marketers utilise numerous persuasive techniques to influence children’s food attitudes, preferences and consumption [4]. Children are especially vulnerable to marketing tactics because their cognitive abilities are still developing [4]. It has been demonstrated that television advertising of unhealthy foods and beverages in South Africa is significantly high [6,7]. Children’s exposure to such adverts influences their preferences and contributes to childhood overweight and obesity, as the products promoted on television are often high in fat, sugar, and salt (HFSS) whilst lacking important nutrients. A systematic review has confirmed that exposure to food and beverage adverts has a direct effect on children’s knowledge, attitudes and how much children who watch a lot of television end up eating [8,9].

The World Health Organization (WHO) has urged governments to implement stringent measures to reduce children’s exposure to the marketing of unhealthy food and beverages [10]. In response, South Africa introduced a voluntary industry initiative known as the Marketing to Children Pledge (the Pledge) which obliged each signatory to ensure that the promotion of healthy dietary choices and lifestyles is achieved, particularly through advertising and marketing communications directed at children aged 12 years or under. The Pledge arose from a collaborative effort between the Department of Health and major food industry actors to limit the marketing of unhealthy foods to children and was developed under the former Advertising Standards Authority (now the Advertising Regulatory Board (ARB)) [6]. It was signed by several leading industry players and set out basic principles intended to guide responsible advertising to children [6]. However, two years after the voluntary pledge was signed, sugar-sweetened beverages (SSBs) were still available in over half of the audited schools and nearly a third continued to advertise them [11].

The framework for regulating advertising in South Africa is fragmented with multiple bodies involved. The framework particularly regulating food and beverage advertising is non-statutory and primarily industry led. The Pledge, alongside the ARB Food and Beverage Code (which closely mirrors the Pledge’s content), illustrates this self-regulatory approach. Industry actors are directly involved in both the design and implementation of these codes, a feature that has prompted debate about the codes’ independence, accountability, and alignment with public health priorities. A growing body of international evidence, particularly from low- and middle-income countries, has found that voluntary commitments by the food and beverage industry consistently fail to achieve meaningful public health outcomes [12]. Despite the voluntary nature of these codes, they have complaint mechanisms, but there is a lack of empirical evaluation of the effectiveness of these mechanisms in the South African context, particularly from the perspective of ordinary consumers. This study investigates the complaint mechanisms available under the current self-regulatory framework in South Africa, focusing on whether they respond to harmful advertising practices – particularly those targeting children with unhealthy food and beverages. It evaluates the timeliness, accessibility, jurisdictional clarity, and procedural and substantive adjudication of the bodies tasked with regulating advertising using the mystery shopper approach. This approach allows assessment of the process as it unfolds in real-world conditions, simulating the experience of consumers who attempt to lodge complaints themselves. Thus, the study seeks to answer the question of whether the existing advertising complaint mechanisms in South Africa are accessible, timely, clear on jurisdiction, and are substantively and procedurally effective in adjudicating complaints of child-directed marketing.

## Background to advertising to children regulation

### General overview

Advertising refers to any paid communication designed to promote goods or services through various media platforms [13]. This includes television, radio, print, websites, social media, and billboards [13,14]. South Africa’s regulatory environment is fragmented, involving several statutory and non-statutory bodies, each with different scopes and complaint mechanisms. The primary regulatory bodies relevant to this study are the ARB; the Independent Communications Authority of South Africa (ICASA); and the Broadcasting Complaints Commission of South Africa (BCCSA). In practice, however, the regulation of advertising content largely lies within the authority of the ARB, a self-regulatory industry body.

Additional mechanisms exist under national legislation such as the Consumer Protection Act 68 of 2008 (CPA) [15] [16] and the Tobacco Products Control Act 83 of 1993 (Tobacco Act) [17]. The Tobacco Act regulates and explicitly bans the marketing or advertising of tobacco products, which also extends to child-directed marketing. However, these were not applicable to the types of adverts identified as relevant for this study, as they did not fall within the respective domains of the CPA or the Tobacco Act.

#### Advertising Regulatory Board (ARB).

The ARB is an industry-funded, self-regulatory body aimed at regulating and upholding ethical standards in advertising in South Africa, ensuring that advertising is “legal, decent, honest and truthful” [18]. This funding structure raises questions about the independence and impartiality of the body, an issue that this study will explore further. The ARB enforces the Code of Advertising Practice (the Code), which includes sector-specific appendices (such as those on food and beverages, and alcohol) and applies across platforms including print, digital, radio, television, and outdoor media [19].

Importantly, the ARB’s jurisdiction is primarily limited to its member organisations, although the Constitutional Court confirmed in *Bliss Brands (Pty) Ltd v Advertising Regulatory Board NPC* (Bliss case) that it may also exercise jurisdiction over non-members who voluntarily submit to its processes [19,20]. Nevertheless, advertisers who choose not to participate may remain beyond the reach of binding self-regulatory decisions, highlighting a key limitation of the current framework. The founding member organisations of the ARB are: Association for Communication and Advertising; IAB South Africa; the Marketing Association of South Africa. Other member organisations are: National Association of Broadcasters (NAB); Cosmetic Toiletry & Fragrance Association of South Africa (CTFA); Drinks Federation of South Africa (DFSA); Association of Independent Publishers; Pet Food Industry Association of South Africa; Vapour Products Association; South African Insurance Association (SAIA) [21].

Complaints must generally be lodged within 90 days and include sufficient information to identify the advertisement [22]*.* A copy of the advert, or details of the time and place of the advertisement must also be submitted in cases where a copy is not available. Although these procedures appear straightforward, this study evaluates how they operate in practice.

#### Independent Communications Authority of South Africa (ICASA).

The ICASA is a statutory body responsible for regulating telecommunications, broadcasting and postal industries [23]. In the advertising context, its mandate is limited to scheduling and placement of adverts (such as infomercials or sponsorships) rather than their content. Complaints about misleading advertising or misrepresentation fall outside its remit [24]. All complaints should be lodged via email.

#### Broadcasting Complaints Commission of South Africa (BCCSA).

The BCCSA functions as an independent tribunal recognised under the ICASA and has jurisdiction over broadcasters who are members of the National Association of Broadcasters (NAB) [25]. It administers codes governing free-to-air, subscription, and online broadcasting services. These codes regulate programming standards, including child protection measures such as watershed periods and restrictions on harmful content [26].

Complaints must be submitted within 30 days of the broadcast via email or postal services [27]. Once accepted, the BCCSA may request the relevant material and a formal response from the broadcaster [27].

#### Outdoor Advertising By-laws – City of Johannesburg.

Outdoor advertising in South Africa is governed by municipal by-laws, which vary across jurisdictions. Municipal by-laws, particularly those governing outdoor advertising in Johannesburg, are relevant to this study. The City of Johannesburg’s by-laws regulate the form, location, and approval of outdoor advertising such as billboards and posters near schools, sports fields, and public facilities [28]. However, the greatest limitation of the by-laws is that they do not regulate the content of adverts but rather focus on the size and location of the billboard/poster [29]. This ultimately leaves the regulation of the content of adverts to the ARB’s self-regulatory mechanism. Furthermore, the by-laws do not provide any public complaint mechanisms and instead focus on penalties for non-compliance. This gap means that the regulation of outdoor advertising content ultimately depends entirely on the ARB’s self-regulatory mechanism. In practice, the regulation of advertising content in South Africa largely lies with the ARB, highlighting the voluntary nature of the current framework.

## Methods

### Ethics statement

The study received ethical approval from the Human Research Ethics Committee (Non-Medical) at the University of Witwatersrand under protocol number H23/03/12. The study was conducted in accordance with the conditions of the approved research protocol. Identifying information relating to individual complainants and individuals encountered through the complaint processes was treated confidentially and not reported in the manuscript unless necessary for the presentation of the findings. The study did not seek to assess or report on the conduct or characteristics of individual complainants but rather examined the functioning and outcomes of the complaint mechanisms.

### Study design

This study used an observational case study design, incorporating a “mystery shopper” approach to assess the procedural and substantive effectiveness of advertising complaint mechanisms in South Africa. The mystery shopper methodology is an accepted research practice where observation of a process without prior consent from a body/person is the only mechanism to obtain valid data [30]. The methodology is frequently used in public health settings [31] and works well when comparing different fora/study sites [32], as it can counteract selection bias where certain individuals might be more prone to use services in a certain way [33]. This method was used because the study aimed to evaluate how complaint mechanisms function in real world conditions when accessed by ordinary members of the public. Researchers therefore acted as complainants and followed publicly available complaints procedures without notifying the regulatory bodies in advance.

### Study setting and platforms

The study was conducted in Johannesburg, South Africa. Johannesburg was selected as it is a densely populated urban hub with widespread outdoor advertising, making it an appropriate location to assess the visibility of advertisements in everyday life. Four advertising platforms were included: outdoor advertising (billboards and posters), radio (Metro FM and Ukhozi FM), television (SABC 1, streamed online on SABC Plus) and YouTube which is the most widely used social media platform in South Africa [34,35]. These platforms were selected based on high national viewer and listenership. Outdoor advertising was included due to its widespread visibility and exposure [36]. Johannesburg is a dense urban hub with extensive billboard advertising, and studies show that approximately 75% of South African consumers reported noticing outdoor advertising in one week, with high levels of message recall and brand engagement [36].

### Data collection protocol

Data collection was conducted in two rounds: April-May 2024 and July-August 2024. The second round focused exclusively on radio and was prompted by the dispute of a complaint submitted during the first round, which required additional observation of comparable radio advertising.

Researchers followed a structured observation protocol. For each advert observed, the following information was recorded in a spreadsheet: platform, date, time, location where relevant, programme or content being viewed or heard where applicable, product or service advertised, brand, category of advert, potential public health concern, likely regulatory body, and whether sufficient information was available to lodge a complaint. In all cases, the researchers acted as ordinary members of the public, observing or listening to adverts in publicly accessible settings without interacting with the platforms. No screenshots, recordings, or downloads were made. This approach was intentionally adopted to replicate the evidentiary limitations faced by ordinary complainants engaging with advertising complaints in real time.

Outdoor adverts were captured within a 5km radius of three purposively selected Johannesburg primary schools representing different socio-economic contexts, based on the 2020 Cooperative Governance and Traditional Affairs Profile [37]. Television, radio, and YouTube were monitored during typical child-viewing/listening hours, i.e., 06h00-08h00; 13h00-15h00 and 15h00-17h00 on weekdays, and 13h00-15h00 on weekends. YouTube data were collected by streaming children’s programming during these times.

A total of 112 adverts were captured across the four platforms. These adverts formed the broader observational sample from which complaints were selected.

#### Classification of adverts.

All captured adverts were categorised into one of three categories using operational definitions based on potential public health concerns and regulatory clarity, which were developed before complaint submission:

- Unproblematic adverts were adverts with no apparent contravention of any existing public health code, by-law, regulation, or policy. These were included to test whether complaint mechanisms could identify and dismiss complaints where no clear violation was present.
- Grey areas adverts were adverts where public health concerns were present, but the regulatory position was uncertain. This included adverts for unhealthy foods or beverages where the product was potentially harmful from a public health perspective, but existing South African regulation did not clearly prohibit the advert. Grey area classification therefore reflected regulatory ambiguity rather than disagreement between researchers.
- Problematic adverts were adverts with an apparent contravention of an existing code, by-law, policy, or regulation, or where the advert raised a clear child-protection concern (the highest number sampled). This included alcohol advertising near schools or during times when children were likely to be exposed.

Where classification involved unhealthy food or beverage advertising, researchers considered the ARB’s Food and Beverage Code and the draft Regulation R3337, which will require warning labels on foods high in salt, unhealthy fats, sugar and non-nutrient sweeteners, and impose child-directed marketing restrictions on products carrying a warning label. While South Africa’s draft Nutrient Profile Model (NPM) introduced under the proposed Regulation R3337 currently only applies to pre-packaged food and beverages, its underlying principles were used to inform the classification of the advertised products. Although the South African NPM remains in draft form, its principles align with internationally recognised models such as those developed by WHO and PAHO, which provide objective criteria for nutritional assessment. In the absence of a binding national framework for fast food, international public health nutrition criteria and established NPMs, including those developed by WHO and PAHO, were also consulted to determine whether featured products, such as pizza bundled with sugar-sweetened beverages (SSBs), could reasonably be classified as unhealthy.

#### Selection of adverts for complaints testing.

From the 112 adverts captured, nine adverts were purposively selected for complaint submission. Selection was guided by the study’s analytical aim of testing how complaint mechanisms respond across varying levels of regulatory clarity, rather than to produce a statistically representative sample.

Adverts were selected to ensure representation across the three predefined classification categories (unproblematic, grey area, and problematic) and across platforms, with priority given to adverts that most clearly exemplified each category based on the operational definitions. Problematic adverts were prioritised to test responses to apparent violations, while grey area and unproblematic adverts were included to assess responses to regulatory ambiguity where no violation was evident.

The final analytical sample included five problematic adverts, two grey area adverts, and two unproblematic adverts. This distribution reflects the study’s focus on evaluating complaint mechanisms under conditions of both regulatory clarity and uncertainty.

Although nine adverts were selected, eleven complaints were lodged because two adverts were submitted to more than one regulatory body to assess jurisdictional clarity, referral practices, and inter-agency coordination. Accordingly, the complaint, rather than the advertisement, constituted the primary unit of analysis throughout the study.

### Complaint submission procedure

Four generic email accounts were created to simulate ordinary members of the public. Complaints were submitted using the procedures published by each relevant regulatory body, including five to the ARB, three to the BCCSA, two to the City of Johannesburg, and one to ICASA. Where no response was received, follow-up correspondence was initiated within two weeks.

The relevant complaints body was selected based on the platform, product, and apparent jurisdiction. Complaints about advertising content were submitted to the ARB where its Code appeared applicable. Complaints involving broadcast-related issues were submitted to ICASA or the BCCSA. Outdoor advertising complaints were submitted to the City of Johannesburg because municipal by-laws regulate outdoor advertising placement and approvals.

Complaints were classified as either correct referrals or incorrect referrals. Correct referrals were complaints submitted to the body that appeared to have formal jurisdiction. Incorrect referrals were deliberate submissions to bodies with limited or no jurisdiction used to assess whether ordinary complainants would be redirected clearly and accurately.

No appeals were lodged because the study was designed to evaluate the initial complaint experience of ordinary complainants rather than the full adjudicative process. This approach reflects the reality that many members of the public may not have the time, knowledge, or resources to pursue appeals following an initial decision. Accordingly, the study focused on accessibility, procedural responsiveness, jurisdictional clarity, and the substantive handling of complaints at first instance.

### Data management

All study data, including information extracted from advertisements, complaints, regulatory correspondence, follow up communications, response dates, outcomes, and reasons provided by regulatory bodies, was recorded in a structured spreadsheet. The data was coded using a predefined coding framework (S1 Table).

Identifying and confidential information is not included in the publicly available supplementary material. Original correspondence was retained separately from the coded analytical data. Some of the correspondence may contain personal identifiers or other confidential information, and access to the original correspondence is restricted to authorised members of the research team.

Data used for analysis and reporting was handled in accordance with the requirements of the study’s ethical approval and applicable confidentiality and data protection requirements. The research data is stored on the principal institution’s password protected storage system, with access restricted to authorised staff of the institution.

### Coding and analysis

The analysis combined descriptive procedural assessment with structured qualitative coding of complaint outcomes. Each complaint was analysed as a separate analytical unit, including where multiple complaints related to the same advert.

A structured codebook guided all data extraction and coding of selected adverts and their associated complaint outcomes (S1 Table). The codebook included predefined variables across four domains: 1) advert characteristics and classification; 2) complaint submission and routing; 3) procedural outcomes; and 4) substantive assessment. Explicit decision rules were developed to guide the classification of adverts as unproblematic, grey area, or problematic, based on regulatory clarity and the presence of public health concerns.

Procedural effectiveness was assessed using standardised indicators including acknowledgement of complaints, response time, number of follow-ups required, clarity and accessibility of the process, evidence requirements, referral accuracy, and whether the matter was resolved. Substantive effectiveness was assessed based on whether the relevant body accepted jurisdiction, identified and applied applicable codes or rules, provided reasoned decisions, and engaged with child-directed marketing, child exposure, or broader public health concerns. Procedural and substantive dimensions were analysed together as they are closely interlinked in shaping the overall effectiveness of advertising complaint mechanisms. This approach enabled the identification of both systemic issues and trends specific to individual regulatory bodies in how complaints are managed and resolved.

To ensure consistency and reduce subjectivity, predefined rating thresholds were applied to classify procedural and substantive effectiveness as satisfactory, partially satisfactory, or unsatisfactory. These thresholds were based on predefined criteria, including response timelines, need for follow-up, completeness of responses, and the extent of regulatory engagement (S1 Table).

Coding was conducted by the lead researcher using the structured codebook. A second researcher independently reviewed the coding framework, a subset of coded cases, and the interpretation of key findings to support consistency and credibility. Any discrepancies or uncertainties were discussed and resolved through consensus. To minimise bias, coding was guided by predefined decision rules, independently reviewed by a second researcher, and discrepancies were resolved through consensus, alongside reflexive consideration of the researcher’s positionality.

## Results

### General observations

A total of 112 adverts were captured across four platforms during both the data collection periods. As shown in Table 1, this descriptive overview contextualises the complaints tested in subsequent sections.

**Table 1:** Distribution of advertisements captured by category and platform.

Categories of adverts captured	Outdoor	Television	Radio	YouTube	Total
Unhealthy food	10	3	5	0	18
Healthy food	0	1	0	0	1
Sports related	0	0	2	0	2
Gambling related	0	0	3	0	3
Insurance	0	0	4	1	5
Mobile network	0	3	3	1	7
Vehicle related	0	0	4	3	7
Appliances	0	0	0	1	1
Cosmetics and body products	0	6	2	5	13
Medicines	0	0	1	2	3
Alcohol	3	0	3	0	6
Political	0	0	2	0	2
Cleaning appliances/products	0	5	1	0	6
Food delivery services	0	3	0	0	3
Food stores	0	3	3	0	6
Finance/banking related	0	0	2	1	3
Broad category of other promotional adverts	0	0	16	10	26
Total number of adverts captured	**13**	**24**	**51**	**24**	**112**

This overview illustrates the marketing environment that children are routinely exposed to, providing the backdrop for assessing the effectiveness of existing complaint mechanisms. The majority of adverts were observed on radio (n = 51), followed by television and YouTube (both n = 24). Unhealthy food and beverage advertisements comprised a significant portion (n = 18) and was concentrated in outdoor advertising, television and radio platforms, while alcohol-related advertising (n = 6) appeared exclusively on outdoor and radio platforms. Both categories are of relevance given their potential impact on children and their positioning within existing and emerging regulatory frameworks.

Of the nine adverts selected for complaints testing, five were classified as problematic (including alcohol billboards located within 700 metres of schools and three alcohol adverts aired on radio during child-listening hours). Two advertisements were classified as grey areas (television adverts promoting fast food and sugary beverages (SSB) combinations). Two were classified as unproblematic (a radio advert promoting safe sex and a YouTube advert promoting AI services). These nine adverts resulted in 11 complaints in total, as some cases were submitted to multiple agencies to test inter-agency coordination. These cases are unpacked in the sections below.

### Correct referrals

#### Outdoor advertising.

Two alcohol-related adverts on billboards were reported to the City of Johannesburg on 13 May 2024 by email. Both billboards were located within 700 meters of schools. Procedurally, four follow ups were made over two and a half months, including three emails and one phone call. No response was received by 31 May 2025. Thus, no substantive assessment or enforcement action was undertaken.

#### Radio.

Two alcohol adverts were reported to the ARB. The first aired on Ukhozi FM on a Saturday at 13h58, was submitted on 17 July 2024 and initially received an automated acknowledgement. A follow-up was made twelve business days later after no further response was received. Thirteen business days later, the ARB rejected the complaint, claiming it fell outside the 90-day timeframe. After clarification that the complaint was within the timeframe, the ARB assessed the matter and concluded the advert complied with its Code. The reasoning provided was that alcohol advertising is allowed on weekends after 12h00, and that it depended on the programming around the advert, and whether or not it was specifically designed to appeal to underage listeners. They continued to state that given that time had passed, it was difficult to ascertain what programming it was. The email further stated that adverts designed to appeal to children are to be reported.

The second complaint, lodged on 20 August 2024, also involved a Saturday afternoon alcohol advert aired on Ukhozi FM at 13h05. The ARB responded the next day seeking confirmation of date and time. In response, we clarified that the advert aired at 13:05 and noted that the ARB complaints form does not permit the entry of 5-minute intervals, potentially limiting the precision of time-stamping adverts. Substantively, the ARB responded 10 business days later and concluded that the advert complied with the Code (Clause 3.3.1.4 of Annexure A), which permits weekend alcohol broadcasts between 12h00 and 06h00.

In both cases, the ARB engaged procedurally but ultimately ruled that the adverts were compliant.

#### Television.

Two adverts promoting fast food bundled with SSBs during child-viewing hours (aired on a Tuesday at 06h13 and 06h50) were reported to the ARB on 10 June 2024 and acknowledged on the same day. Procedurally, eight business days later, the ARB relayed that the broadcaster could not locate the adverts and had confirmed that they were not aired, suggesting the complaint time was inaccurate. We double checked and reconfirmed the correct time (06h13 and 06h50) on 26 June. However, this went to the ARB’s junk mail delaying the process until early August. After one follow up, the ARB’s substantive assessment concluded (thirteen business days later) that like alcohol and gambling, it is not illegal to advertise fast food per se and that it can only assess how a product is advertised. It found that the adverts did not appear to contain anything that contravened the Code’s principles.

Procedurally, the complaint was delayed given that our email was misdirected to the ARB’s junk folder and proceeded only after a follow-up prompted retrieval. Substantively, it was concluded that the adverts did not contravene the Code.

#### YouTube.

An advert promoting AI services was reported to the ARB on 12 August 2024 and acknowledged the same day. The next day, the ARB requested further evidence, noting that without a link or screenshot, it was unclear whether the insert constituted an advert. Sixteen business days later, the ARB reiterated that they could not proceed without this evidence and closed the file.

Procedurally, the ARB engaged promptly, but the complaint did not proceed to substantive assessment because no link or screenshot was provided.

### Incorrect referrals

Two adverts relating to alcohol were reported to the BCCSA on 13 May 2024, to test their response to an incorrect complaint. On the same day, the BCCSA responded, clarifying that it only considers complaints regarding the content of broadcast programmes, not adverts, and referred the matter to the ARB, which was the correct forum.

An advert promoting condom use was reported to ICASA on 17 May 2024. ICASA acknowledged receipt three business days later on 22 May 2024. Following its investigation, ICASA found (the same day) that it did not have jurisdiction and referred the complaint to the BCCSA. We then sent the referral to the BCCSA on 27 May 2024 and received a response the same day noting that ICASA’s referral was incorrect and redirected the complaint to the ARB, which was the appropriate forum.

Procedurally, both the ICASA and BCCSA responded promptly, but substantively the responses were unsatisfactory given the incorrect referral by ICASA and the BCCSA’s inability to consider the complaint. Table 2 presents a pictorial summary of the timelines for each advert reported.

**Table 2:** Summary of the timelines for each advert reported.

Regulatory Body	Product	Platform	Days taken for Resolution	Outcome	Follow-Up Required	Procedural (P) or Substantive (S) Notes
ARB	Alcohol	Radio	19 days	Weekend alcohol adverts allowed after 12h00; restrictions apply if content appeals to underage listeners.	Yes	(P) Unsatisfactory, follow up required; time-frame dispute not justified. (S) Unsatisfactory, potential gap in Alcohol Appendix watershed period.
ARB	Alcohol	Radio	15 days	Adverts complied with Clause 3.3.1.4 – aired within permitted weekend broadcast hours(12h00-06h00).	No	(P) Unsatisfactory, assessor requested exact airtime, but ARB form does not allow for 5-minute intervals. (S) Unsatisfactory, potential gap in Alcohol Appendix watershed period.
ARB	Unhealthy food	TV	59 days	ARB only assesses advert content for Code breaches; product type (pizza, Coke), not prohibited.	Yes	(P) Unsatisfactory, lengthy process, issue with sourcing advert and further delay from email going to junk mail. (S) Highlighted regulatory gaps for unhealthy food marketing during child-viewing times.
ARB	Promo Ad - AI	YouTube	18 days	Investigation could not proceed without link or screenshot; file closed.	No	(P) Satisfactory. (S) Unsatisfactory, ARB’s Code does not require a link or screenshot.
ICASA	Promo Ad – Safe sex	Radio	2 days	No jurisdiction – referred to BCCSA.	No	(P) Satisfactory. (S) Unsatisfactory, incorrect referral.
BCCSA	Alcohol	Radio	1 day	Only considers broadcast programme content, not ads; referred to ARB.	No	(P) Satisfactory. (S) N/A.
BCCSA	Promo Ad – Safe sex	Radio	1 day	BCCSA noted complaint; ICASA referral incorrect; redirected to ARB.	No	(P) Satisfactory. (S) N/A.
COJ	Alcohol	Outdoor Advertising	1 year	No response as of May 2025.	Yes	(P) Unsatisfactory, no response received to date. (S) N/A.

Note: P = Procedural; S = Substantive.

## Discussion

### Procedural efficiency and accessibility of complaint mechanisms

By employing a mystery shopper approach, we uncovered procedural barriers that may remain hidden in policy-level evaluations, demonstrating that the accessibility of complaint mechanisms depends not only on the existence of procedures but also on how they operate in practice. The marked variation in responsiveness across regulatory bodies, including the absence of any response from the City of Johannesburg, illustrates substantial inconsistencies in procedural accessibility. The ‘silence’ of a government agency represents the most severe procedural barrier, effectively closing off any avenue for complaints and leaving outdoor advertising content regulation solely in the hands of self-regulation.

Although the City of Johannesburg’s Outdoor Advertising by-laws do not govern advert content, they are responsible for approving, monitoring, and placement of adverts. Placement restrictions, such as proximity limits to schools, are integral to reducing children’s exposure. While the ARB would assess content, municipalities have a parallel role through by-laws and should at the very least acknowledge the complaint and say it is not within their jurisdiction. Further research is needed to determine if similar gaps exist in other cities. These findings point to the need for a centralised body to manage complaints, refer matters to municipalities, and follow up. Given that most reported adverts were for unhealthy food, this procedural gap is especially concerning in the context of unhealthy food advertising, an area where South Africa’s substantive regulatory framework remains limited despite the proposed restrictions under R3337 [6,38,39].

Repeated follow-up requirements, particularly with the ARB, created unnecessary procedural barriers for complaints. In one instance, our email was directed to the ARB’s junk mail folder, meaning they were unaware of the correspondence until we followed up. Another issue was that the ARB’s online form could not capture exact times in five-minute intervals, limiting precision in timestamping and sourcing adverts. In one case, the ARB first disputed the timeframe and then stated that too much time (70 days) had passed to identify the programme context of an advert despite complaints being accepted within a 90-day window, suggesting inadequate mechanisms for retrieving and assessing broadcasts within their own procedural timeframe. Given the 90-day window, retrieval mechanisms are essential as members of the public cannot always report the advert immediately. The ARB also required links or screenshots of adverts before proceeding, despite Clause 3.1.4 of its procedural guide not making this mandatory if sufficient descriptive detail is provided. Given that detailed information about the advert was provided, it is unclear why the ARB could not proceed with the investigation. This requirement, although not mandated in its procedural guide, creates an unnecessary procedural barrier, particularly when capturing such evidence is impractical (e.g., when a complainant is driving or otherwise unable to record the advert at the time it airs). Of further concern is that in one instance, the ARB appeared to automatically accept the broadcaster’s denial that an advert had aired, once again placing the onus on the complainants to prove otherwise.

These procedural hurdles risk discouraging complaints, particularly from members of the public who lack time or technical means to document adverts immediately and follow up regularly. In our study, none of the problematic complaints were resolved satisfactorily, and in some cases, it took multiple follow-ups and substantial effort just to receive a response. For members of the public, the process is particularly onerous, placing the onus on complainants to provide detailed evidence and persist through repeated delays or incorrect referrals.

### System fragmentation and jurisdictional uncertainty

The complaints process was also hampered by fragmentation across regulatory bodies, which is not merely a structural flaw. It imposed a significant burden on complainants by requiring them to navigate overlapping and sometimes poorly understood jurisdictions. Incorrect referrals further prolonged complaint resolution and undermined accessibility.

In outdoor advertising cases, the ARB shares jurisdiction with municipalities. While the municipalities have specific by-laws, they were unresponsive in our study, effectively leaving the ARB as the only active forum. This concentration of responsibility in a self-regulatory body, combined with the procedural inefficiencies outlined above, reinforces the need for a centralised co-regulatory body involving shared oversight between government and industry with statutory backing, or preferably a fully statutory model with legally mandated government oversight. WHO and global research has evidenced that self-regulation of food and beverage marketing has failed to produce meaningful outcomes in reducing children’s exposure to unhealthy food and beverage advertising globally [40,41]. International experience supports the advantages of more centralised statutory or co-regulatory approaches, particularly in reducing fragmentation, improving jurisdictional clarity, and enabling more coordinated oversight across television, schools, social media, packaging, outdoor, and radio platforms. Such regulatory approaches also provide stronger enforcement mechanisms for non-compliance [42–44]. For example, Chile’s statutory Food Labelling and Advertising Law introduced broad restrictions across televisions, school, packaging, and promotional giveaways, contributing to reduced exposure to unhealthy food marketing [45,46]. Similarly, the United Kingdom’s co-regulatory framework combines statutory nutrient profiling with oversight by both government and the advertising regulator across broadcast and digital media has been adopted [42].

### Substantive outcomes and the limits of self-regulation for child protection

#### Weekend broadcast rules: Inconsistencies with child-protection aims.

Although the study primarily evaluated complaint mechanisms rather than substantive advertising regulation itself, the adjudicative outcomes revealed important limitations in the standards applied by complaints bodies which is equally important from a regulation and child protection perspective. The ARB’s response to the alcohol adverts reported to them is concerning not because the ARB misapplied its rules but because the rules themselves are poorly designed. For example, the ARB applied Clause 3.2.4.4 of the Alcohol Code, which states that alcohol adverts may be broadcasted between 12h00 and 06h00 on Saturdays and Sundays. As written, the Code permits alcohol adverts from midday to 06h00 the following day on weekends, creating a substantially broader broadcasting window than during weekdays. This is difficult to reconcile with child-protection objectives, given that children are more likely to be watching television or listening to radio on weekends when they are not at school. In practice, the weekend watershed should arguably be shorter, not longer – perhaps starting at 21h00 when many children are asleep.

The watershed period is also inconsistent when compared to other protection clauses such as Clauses 3.2.1 (no adverts immediately before, during, or after children’s programmes), Clause 3.2.2 (no adverts in media explicitly aimed at children), and Clause 3.2.3 (no adverts in programmes with a 30% or more underage audience). These provisions show a stated intention to limit exposure, yet the weekend broadcasting window in Clause 3.2.4.4 undermines that objective.

#### Outdoor alcohol advertising: Loopholes in distance-based restrictions.

The outdoor advertising complaints also illustrate the limitations of distance-based placement restrictions. Even where advertisements complied with the prescribed 500 m threshold, children may still be exposed while travelling to and from school [47]. Such tactics highlight the limitations of distance-based restrictions and strengthen the case for a complete prohibition on outdoor alcohol advertising near spaces frequented by children, or ideally, a comprehensive ban on outdoor alcohol advertising altogether (as implemented in Lithuania) [48].

#### Limits of the “child-directed” test and asymmetric standards in self-regulation.

The ARB Food and Beverage Code applies its child-directed marketing provisions only to children under 12, which significantly limits its protective scope. From our findings, this narrow age focus is compounded by the ARB’s frequent reliance on whether the advert was “specifically designed to appeal” to underage audiences, overlooking evidence that mere exposure can influence consumption patterns [49]. This narrow approach also fails to account for adolescents, despite evidence that exposure to marketing influences older children and teenagers differently from younger children [50–53]. The watershed model also assumes that children are not viewing outside regulated hours, which is unrealistic for adolescents and older children, especially where parental oversight is limited. This is a major oversight given that they are also vulnerable to marketing tactics and need to be protected from harmful products [54].

The ARB’s interpretation in the Fair Cape Dairies case illustrates this narrow approach. The complaint involved a chocolate flavoured milk packaging featuring Barbie imagery, which the complainant argued directly appeals to children under 12. The ARB accepted the advertiser’s nutritional framing and found the packaging compliant with the Code [55,56]. This is despite the fact that the product contains 36.6g of sugar, which is well above the maximum recommended daily amount for a young child, and approximately 1.5 to 3 times over the WHO guideline, depending on the threshold. This reasoning prioritised whether the advert was explicitly directed at children rather than considering the broader persuasive effect of child-appealing imagery and exposure. The ARB’s reasoning illustrates how self-regulatory forums’ reliance on an advertiser’s framing may limit consideration of the broader child-protection implications of child-appealing imagery and exposure.

Past ARB decisions suggest that broader exposure-focused interpretations are possible. In Dotsure – du Plessis [57] and First for Women – Karen Feuth & Others, the ARB acknowledged that adverts should consider the impact of adverts on younger viewers even where children were not the intended audience [58]. These precedents contrast with the narrower approach observed in the present study.

In contrast, a public health “radio commercial” by the Healthy Living Alliance supporting South Africa’s SSB tax was found misleading for stating that SSBs cause heart disease without qualification [59]. This decision is concerning for two reasons. First, the public health civil society organisation appears to have been held to a higher evidentiary standard than the advertisers of the chocolate milk, raising concerns about whether industry and public health communications are assessed consistently. Second, the breadth of Clause 4.1.2’s definition of “advertisement” is questionable, as it extends full advertising restrictions to cause-based public interest communications. Colombia’s Constitutional Court dealt with a similar issue pertaining to censorship on an obesity prevention campaign, which was banned. The Court ruled the ban violated consumers’ rights to know about the harms [60].

These contrasting outcomes, one permitting a child-appealing product based on a narrow reading of “child-directed”, and another restricting a public health message on evidentiary grounds, suggest possible asymmetries in how industry and public health communications are assessed. They also raise broader concerns about the fairness and perceived impartiality of self-regulation, given that the ARB is funded by marketers and advertisers, many of whom are the subjects of complaints brought before it. The ARB Funders are: ABSA Bank Group; Allan Gray; Ardagh Group; Clicks; Clientèle Limited; Coca Cola; Colgate-Palmolive; Debonairs Pizza; Discovery; Fishaways; First National Bank (FNB); Gendel Advertising; Hollard; Hunt Lascaris; Investec; Kellanova; KFC; Luno; Melbro Brands; MTN; Mugg & Bean; MWeb; National Association of Broadcasters; Nandos; Nedbank; OUTsurance; Procter & Gamble; PepsiCo; Pick n Pay; Professional Provident Society (PPS); Premier FMCG; Reckitt.com; RCL Foods; Sanlam; Standard Bank; Steers; Suzuki; TBWA/SA; Tiger Brands; Unilever; Vodacom; and Wimpy [21].

These findings, considered alongside the broader literature, raise legitimate concerns about the fairness, impartiality, and objectivity of the self-regulatory mechanism and may help explain the asymmetry in the outcomes observed in these cases. These broader examples are consistent with the procedural and substantive patterns observed in the present study, including narrow application of the “child-directed” test, where exposure risks were overlooked. They are also indicative of procedural asymmetries where greater weight was given to advertiser statements such as accepting an assertion that an advert was not flighted without requiring proof, despite the practical challenges complainants face in recording content, which is also not a mandatory requirement. This asymmetry may reflect structural conflicts of interest inherent in self-regulation, where the regulator is funded by the very industry it is tasked with overseeing. Concerns about the limitations of self-regulation are not unique to South Africa, with studies in other settings reporting limited effectiveness in reducing children’s exposure to unhealthy food advertising. A global study found that children in countries with self-regulatory advertising codes were exposed to more unhealthy food advertisements than those in countries with no regulations [61]. In Canada, industry self-regulation produced minimal change in children’s exposure to unhealthy food advertising [62]. In contrast, jurisdictions like Chile have moved toward mandatory statutory controls, which cover more media platforms and impose stricter nutrition standards, offering a more effective regulatory framework [63]. These findings align with global evidence highlighting that industry voluntary codes often fail to reduce exposure to unhealthy food advertising whereas statutory regulation and bans have delivered significant reductions in exposure.

### Strengths and study limitations

A key strength of this study lies in its mystery shopper approach, which generated real-world evidence on how advertising complaints are processed in South Africa. The approach helped uncover invisible barriers that ordinary consumers face, barriers which would remain hidden in purely paper-based policy assessments. Linking procedural analysis with substantive case outcomes enabled the study to identify limitations in the design or application of the rules and examine how features of the system may affect accessibility. Another key strength of the study was the choice to observe outdoor adverts in areas with diverse socio-economic contexts, enhancing both the credibility of the findings and their capacity to reflect the broader urban environment.

However, several limitations should be noted. First, the study was conducted exclusively in Johannesburg. The limited geographic scope may constrain the generalisability of the findings as regulatory practices, administrative responses, and enforcement capacity may vary across municipalities and provinces. However, Johannesburg is South Africa’s largest metropolitan municipality and economic hub, making it an important setting for examining the operation of advertising complaint mechanisms. In addition, one of the four regulatory bodies approached during the study, the City of Johannesburg, did not respond to any complaints submitted during the study period. While this limited our ability to assess municipal complaint handling beyond procedural responsiveness, the absence of a response itself constitutes an important finding regarding the responsiveness of the current regulatory framework. Future research across additional jurisdictions would help determine the extent to which these findings are observed nationally.

Second, the study did not evaluate appeal processes. This reflects the methodological choice to examine the initial complaint experience of ordinary complainants rather than the full adjudicative process, recognising that many members of the public may not pursue appeals because of the additional time, knowledge, and resources required. Consequently, the findings relate to first-instance complaint mechanisms and do not assess whether appeal processes may improve procedural fairness, accountability, or substantive outcomes. Future research should examine appeal processes to determine whether initial decisions are upheld, modified, or overturned, and whether appeals address the procedural and substantive shortcomings identified in this study.

Third, this study provides a snapshot of complaint handling during the study period. The response time and procedural experiences observed may not reflect longer-term administrative performance or changes in regulatory practice over time because data were collected through real-time complaint submissions.

Fourth, classification of unhealthy products was informed by adapted NPM principles for fast food. Although these principles are consistent with internationally recognised WHO, PAHO models, they are not yet formally embedded within South African regulation.

Finally, although the qualitative observational case study design provided rich insights, the purposive sampling and limited number of complaints restrict statistical or population-level generalisability. Nonetheless, purposive sampling enabled assessment across multiple regulatory bodies, advertising platforms, and varying levels of regulatory certainty, allowing the study to examine complaint mechanisms under diverse real-world conditions.

### Recommendations

The procedural and substantive shortcomings identified in this study, when considered alongside the broader literature on advertising self-regulation and marketing to children, suggest important limitations in the current framework [64–69]. The findings suggest that stronger co-regulatory or statutory approaches may be necessary to address the procedural and substantive limitations identified in the current self-regulatory framework. While the ARB is presently the primary assessor of advertising content, the following reforms are needed to strengthen the framework and protect children from harmful marketing.

1. Improve complaints procedures
  - A centralised complaint mechanism could be established to triage complaints to the correct body, reducing jurisdictional confusion and delays. The centralised complaints body should be a statutory authority, to ensure independence and enforcement power.
  - The ARB should amend procedures to allow investigations based on sufficiently detailed descriptions of adverts, and strengthen its retrieval mechanisms for adverts, rather than require screenshots or links which places a higher evidentiary burden on complainants than on advertisers even though they are not currently mandatory. Advertisers, not complainants, should bear responsibility for retaining and providing advertising evidence when requested.
  - The general complaints system should be made user-friendly, with safeguards to prevent delays caused by technical oversights (e.g., emails going to junk mail or 5-minute interval limit on advert timestamping) and with reasonable response times that do not rely on complainants to initiate repeated follow ups.
2. Strengthen alcohol marketing restrictions
  - Proximity limits in the Alcohol Code should be extended beyond 500 m or replaced with a complete prohibition on outdoor advertising near spaces frequented by children, or ideally, a comprehensive ban (as implemented in Lithuania).
  - Weekend broadcast hours for alcohol advertising should be reduced to 21h00-06h00 to align with child-protection aims or replaced with a complete ban for alcohol and other harmful products such as unhealthy foods. WHO and PAHO endorse restrictions on alcohol marketing [70–72]. Broad bans on alcohol advertising that cover all advertising, sponsorship, and promotions across traditional and digital platforms have been shown to effectively reduce drinking among young people [73]. Research indicates that limiting youths’ exposure to messages portraying alcohol as linked to social success or positive lifestyle values can delay the age at which they begin drinking and lower the amount they consume [74].
3. Align definitions and scope with child-protection standards
  - The ARB Food and Beverage Code currently limits protections to children under 12. This should be expanded to protect all persons under 18, in line with South Africa’s legal definition of a child and WHO recommendations.
  - The Code should adopt an exposure-based standard rather than relying solely on whether advertising is “specifically directed” at children. This would close a significant gap by recognising that mere exposure has measurable impacts on adolescents, in line with global public health evidence.
  - The ARB’s definition of “advertisement” should explicitly exclude bona fide public health messaging to avoid unduly restricting cause-based communications.
4. Ensure policy coherence and institutional capacity
  - ARB codes should be aligned with the Draft R3337 which proposes stronger restrictions on unhealthy food and beverage marketing.
  - ARB officials should receive targeted training on public-health oriented advertising regulation including public health and nutrition principles training, alongside technical distinction between child-directed/targeted marketing and exposure. This would equip ARB staff to critically assess industry claims.
  - Once the R3337 is in force, the ARB must develop capacity to handle a potential increase in complaints and apply the new provisions consistently.
5. Address structural conflicts of interest
  - Given that the ARB is funded by marketers and advertisers including those entities potentially subject to its adjudication, independent oversight should be introduced to strengthen fairness, impartiality, and public trust in the complaints process. The ARB could establish an independent oversight committee composed of representatives from public health, civil society, academia, and government, to monitor ARB decisions and strengthen fairness and accountability.

## Conclusion

This study assessed the effectiveness of advertising complaints procedures in South Africa, with a particular focus on protecting children from exposure to harmful product marketing. In the cases examined, the complaints process was cumbersome to navigate and imposed procedural and evidentiary burdens that impeded accessibility and timely resolution. These procedural barriers were closely connected to the substantive handling of complaints and may limit the capacity of existing mechanisms to support the protection of children. The findings also identify limitations associated with reliance on a voluntary, self-regulatory system, including its restricted scope and concerns regarding demonstrably independent and impartial adjudication.

Although the findings derive from a small, purposively selected sample, the weaknesses observed, considered alongside the broader evidence on self-regulation, support stronger government involvement in advertising regulation. A legally enforceable framework, potentially beginning with a co-regulatory model incorporating an independent centralised complaints body, could strengthen accessibility, accountability and the consideration of children’s health in advertising regulation.

## Supporting information

S1 Table Codebook for coding selected adverts and complaint outcomes. Codebook detailing the variables, coding categories, decision rules, and criteria to assess procedural and substantive effectiveness.
(DOCX)

S1 Data Data collection dataset of advertisements captured during the study. Dataset containing the advertisements collected during the study and their associated characteristics.
(XLSX)
