# Supplementary material for: Effectiveness of advertising complaint mechanisms in South Africa: Public health implications and regulatory gaps
Source: PLOS Glob Public Health. 2026 Oct 30;6(10):e0005223. doi: 10.1371/journal.pgph.0005223 (PMC13722151; doi:10.1371/journal.pgph.0005223)
Supplement: Supplementary file 1 [file pgph.0005223.s001.docx]

**Supplementary Table S1: Codebook for coding selected adverts and complaint outcomes**

**Coder instructions:**

- Unit of analysis: Each complaint is the primary unit of analysis linked to a selected advert.
- Where one advert is submitted to multiple bodies, code each complaint separately.
- Coding must be based only on documented observation and correspondence.
- Where information is unavailable, code as “Unknown” (do not infer).
- Complete coding after final response or closure of complaint.

**Section 1: Advert characteristics (descriptive variables)**

| **Variable** | **Description** | **Coding options** | **Decision rule** |
| --- | --- | --- | --- |
| Advert ID | Unique identifier for advert | e.g. A1, A2, A3… | Assign once per selected advert |
| Complaint ID | Unique identifier per complaint | e.g. C1, C2, C3… | Each submission = new ID |
| Platform | Where advert was observed | Outdoor, Radio, Television, YouTube | Select primary platform |
| Product category | Type of product promoted | Alcohol, Unhealthy food and beverage, Tobacco, Other | Code dominant product |
| Brand/company | Brand or advertiser | Free text | Record as observed |
| Observation context | Where/how advert was encountered | Free text | Include programme/location |
| Time of exposure | Time advert observed | 24-hour time | Record as precisely as possible |

**Section 2: Advert classification (analytic variables)**

| **Variable** | **Description** | **Coding options** | **Decision rule** |
| --- | --- | --- | --- |
| Advert classification | Final classification of advert | Unproblematic, Grey area, Problematic | Apply rules below |
| Basis for classification | Primary reasons for classification | Regulatory breach, regulatory ambiguity, public health concern, no apparent issue | Select dominant basis |
| Regulatory clarity | Clarity of applicable rule | Clear, ambiguous, no clear rule | - Clear = binding rule directly applies; - Ambiguous = rule exists but unclear OR draft only; - No rule = no applicable framework |
| Applicable framework | Relevant regulation/code | ARB Code, Alcohol Appendix, Food & Beverage Code, Draft R3337, Municipal by-law, None, Other | Multiple allowed |
| Public health concern present | Whether advert raises public health concern | Yes, No | Includes alcohol or tobacco, unhealthy food, child-directed/appealing |

**Classification decision rules:**

- Unproblematic: No apparent violation AND no significant public health concern
- Grey area: Public health concern present BUT regulation in draft form, unclear, incomplete, or non-binding
- Problematic: Clear or likely contravention of existing rule/code OR strong child-protection concern revealing a potential regulatory gap

**Section 3: Complaint submission**

| **Variable** | **Description** | **Coding options** | **Decision rule** |
| --- | --- | --- | --- |
| Regulatory body | Body complaint submitted to | ARB, BCCSA, ICASA, Municipality | Record actual body |
| Referral type | Whether complaint was correctly directed | Correct, Incorrect | - Correct = body has apparent jurisdiction - Incorrect = body does not have jurisdiction |
| Basis for submission | Why was this body selected | Content regulation, Broadcast issue, Outdoor placement, Jurisdictional test | Select primary reason |
| Evidence available | Information available to complainant | Description only, Time + Date, Location, Link, Screenshot, Other | - Reflect real-world conditions - Multiple allowed |

**Section 4: Procedural outcomes**

| **Variable** | **Description** | **Coding options** | **Coding guidance/decision rule** |
| --- | --- | --- | --- |
| Acknowledgment received | Whether complaint was acknowledged | Yes, No, Automated response only | Automated does not classify as a substantive response |
| Response time | Days to first substantive response | Numeric (days) | Exclude automated replies |
| Follow-up required | Whether follow-up was needed | Yes, No | Yes = no progress without follow-up |
| Number of follow-ups | Total follow-ups | Numeric | Count all attempts |
| Evidence requested | Whether body requested evidence | Yes, No | Record type separately |
| Reasonableness of evidence | Whether request was reasonable | Reasonable, Unreasonable, Not applicable | - Reasonable = evidence was proportionate, consistent with published procedures + realistically obtainable by an ordinary complainant - Unreasonable = not required by procedure OR unrealistic for ordinary complainant |
| Referral made | Whether the complaint was referred to | Yes, No, Not applicable | Record referral destination |
| Referral accuracy | Accuracy of referral | Correct, Incorrect, No response, Not applicable | - Correct = referral directed matter to body with appropriate apparent jurisdiction - Incorrect = referral directed matter to body without appropriate jurisdiction or provided misleading jurisdictional guidance - Not applicable = no referral required |
| Completeness of response | Whether response addressed complaint | Complete, Partial, Incomplete, No response | - Complete = clear outcome + reasoning addressing core complaint - Partial = response addressed some aspects of complaint but omitted important procedural or substantive elements - Incomplete = response provided minimal engagement or failed to address substance of complaint |
| Resolution status | Final status | Resolved, Unresolved, Closed, No response | - Resolved = complaint reached substantive determination or clear procedural outcome - Unresolved = complaint remained procedurally active or outcome unclear at end of observation period - Closed = complaint formally closed without substantive assessment or further action |

**Section 5: Substantive outcomes**

| **Variable** | **Description** | **Coding options** | **Decision rule** |
| --- | --- | --- | --- |
| Jurisdiction accepted | Whether body accepted authority | Yes, No, Not applicable | Record reasoning if declined |
| Code/rule identified | Whether rule cited | Yes, No, Partial, Not applicable | - Yes = specific rule or code clearly identified - Partial = vague reference to rules/codes or incorrect rule cited - No = no rule or code referenced |
| Code/rule applied | Whether rule applied to case | Yes, No, Partial, Not applicable | - Yes = response applied identified rule/code to facts of complaint - Partial = rule/code cited but incompletely, inconsistently, or incorrectly applied - No = no application of rule/code to complaint |
| Reasons provided | Quality of reasoning | Full, Limited, None | - Full = clear logic linking facts + rule - Limited = some reasoning provided but incomplete, unclear, or lacking clear linkage between facts and rule/code - None = no substantive reasoning provided |
| Child-directed marketing considered | Whether exposure assessed | Yes, No, Partial, Not applicable | - Yes = response explicitly engaged with child exposure, audience composition, child-directed appeal, or potential impact on children - Partial = children mentioned but engagement limited, indirect, or incomplete - No = no consideration of child exposure or child-directed concerns |
| Public health concern considered | Whether health issue addressed | Yes, No, Partial, Not applicable | - Yes = response explicitly addressed health-related concerns (e.g. nutrition, alcohol exposure, child health impacts) - Partial = public health issue mentioned but engagement limited or incomplete - No = no engagement with public health concerns |
| Outcome | Final decision | Upheld, Dismissed, Not Assessed, Referred, Closed, No response | Record final outcome as communicated by regulatory body |
| Regulatory gap identified | Whether system limitation revealed | Yes, No, Unclear | Yes = response revealed absence, ambiguity, inconsistency, or procedural limitation in existing regulatory framework or enforcement process  No = existing framework adequately addressed issue  Unclear = insufficient information to determine |

**Section 6: Effectiveness ratings (with thresholds)**

Procedural effectiveness

| **Rating** | **Criteria** |
| --- | --- |
| Satisfactory | Acknowledged + response within stated procedural timelines where applicable or within a reasonable administrative timeframe where no timeline existed + no follow up required + clear process + correct referral (if applicable) |
| Partially satisfactory | Acknowledged but delays relative to procedural timelines (where stipulated), OR follow up required, OR minor procedural barriers |
| Unsatisfactory | No response OR excessive delay, OR incorrect referral, OR repeated follow ups, OR unreasonable evidence burden |

Substantive effectiveness

| **Rating** | **Criteria** |
| --- | --- |
| Satisfactory | Jurisdiction clearly addressed + relevant rule identified and applied + clear reasoning + engagement with child/public health concerns and relevant codes/rules (where relevant) |
| Partially satisfactory | Some reasoning or rule application but incomplete engagement with exposure or public health concerns OR partial application of relevant rule/code |
| Unsatisfactory | No meaningful assessment, OR no reasoning, OR failure to apply rules, OR failure to consider child/public health concern, OR clear regulatory gap |

Section 7: Analytical interpretation

| **Variable** | **Description** | **Coding instruction** |
| --- | --- | --- |
| Key procedural issue | Main procedural barrier identified | Free text (e.g. delay, referral failure, evidence burden)  Multiple allowed |
| Key substantive issue | Main substantive issue identified | Free text (e.g. incorrect application of rule, narrow child-directed test, regulatory gap) |
